# Single-Cell Transcriptomic Analysis Identifies a Translation/RBP-Related Fibroblast Signature for Drug Repurposing in Idiopathic Pulmonary Fibrosis

**DOI:** 10.3390/ijms27219736

**Published:** 2026-10-30

**Authors:** Yongkang Qiao, Chaoyue Cui, Yanhong Han, Xinxin Tang, Zhen Yang, Chuang Ge

**Affiliations:** 1 Center for Biological Science and Technology, Key Laboratory of Cell Proliferation and Regulation Biology, Ministry of Education, Guangdong Zhuhai-Macao Joint Biotech Laboratory, Department of Biology, Faculty of Arts and Sciences, Beijing Normal University, Zhuhai 519087, China; 2 School of Pharmacy, Mudanjiang Medical University, Mudanjiang 157011, China

**Keywords:** idiopathic pulmonary fibrosis, fibroblast, single-cell RNA sequencing, translational regulation, RNA-binding proteins, drug repurposing

## Abstract

Idiopathic pulmonary fibrosis (IPF) is characterized by persistent fibroblast activation and excessive extracellular matrix deposition. However, transcriptional heterogeneity associated with translation- and RNA-binding protein (RBP)-related processes within IPF fibroblasts remains incompletely characterized. Here, we analyzed two independent human IPF single-cell RNA-sequencing cohorts to identify fibroblast states that differed in Translation/RBP-related transcriptional programs while sharing high fibrotic output. Donor-paired pseudobulk analysis in GSE136831 identified a transcriptional signature distinguishing these states, and genes showing consistent directional changes in the independent GSE135893 cohort were retained to derive a cross-cohort signature. Reverse-connectivity analysis across CLUE, L1000CDS^2^, and iLINCS was then used to prioritize compounds predicted to transcriptionally oppose this signature. Candidate compounds were subsequently evaluated in transforming growth factor-β1 (TGF-β1)-stimulated human fetal lung fibroblasts (HFL-1), with cell viability, α-smooth muscle actin (α-SMA), and collagen I used for initial phenotypic assessment. PP-1, PK-11195, dabrafenib, GSK-1059615, and dasatinib reduced both profibrotic markers within the tested concentration ranges, identifying them as candidates for further investigation. Together, these findings define a cross-cohort Translation/RBP-related fibroblast signature in IPF and support its use as a transcriptome-guided strategy for prioritizing compounds with antifibrotic phenotypic activity.

## 1. Introduction

Idiopathic pulmonary fibrosis (IPF) is a progressive and fatal interstitial lung disease characterized by disruption of alveolar architecture and excessive extracellular matrix (ECM) deposition [1,2,3]. Persistent activation of fibroblasts and myofibroblasts promotes sustained production of collagen, fibronectin, and other matrix components, leading to progressive tissue remodeling and loss of lung function [3,4]. Current antifibrotic therapies, including pirfenidone, nintedanib, and the recently introduced phosphodiesterase 4B (PDE4B) inhibitor nerandomilast, can slow the decline in lung function [5,6,7]. Despite these therapeutic advances, IPF remains a progressive disease, highlighting the need for additional strategies to suppress the sustained fibrotic output of activated fibroblasts.

Activated fibroblasts exert their profibrotic effects through sustained production of extracellular matrix and other profibrotic proteins, with post-transcriptional mechanisms such as mRNA stability and translational control shaping the resulting protein output. Translational control has been shown to regulate collagen synthesis and myofibroblast differentiation [8,9,10], while RNA-binding proteins (RBPs) such as HuR and DDX3 have been implicated in fibroblast activation and pulmonary fibrosis [11,12]. However, whether Translation/RBP-related processes collectively define a disease-relevant molecular program in human IPF fibroblasts remains unclear.

Single-cell transcriptomic studies have revealed substantial heterogeneity among fibroblasts in IPF lungs and enabled disease-associated molecular programs to be examined at the level of defined cell populations [13,14,15]. Transcriptome-based connectivity analysis enables the identification of compounds whose perturbational profiles oppose a disease-associated expression signature [16,17,18,19]. Recent studies have further used single-cell-derived transcriptional information to guide drug repurposing [20,21].

In this study, we integrated two independent human IPF single-cell RNA-sequencing cohorts to identify a Translation/RBP-related transcriptional signature in IPF fibroblasts. We then used this signature for reverse-connectivity analysis across CLUE, L1000CDS^2^, and iLINCS to prioritize candidate compounds and evaluated selected candidates in a transforming growth factor-β1 (TGF-β1)-induced human fetal lung fibroblast (HFL-1) activation model. This study establishes a state-guided strategy for linking a Translation/RBP-related fibroblast signature to drug repurposing and antifibrotic screening in IPF.

## 2. Results

### 2.1. Single-Cell Analysis Identifies Translation/RBP-Related Transcriptional States and a Transcriptional Signature in IPF

**Figure 1:**
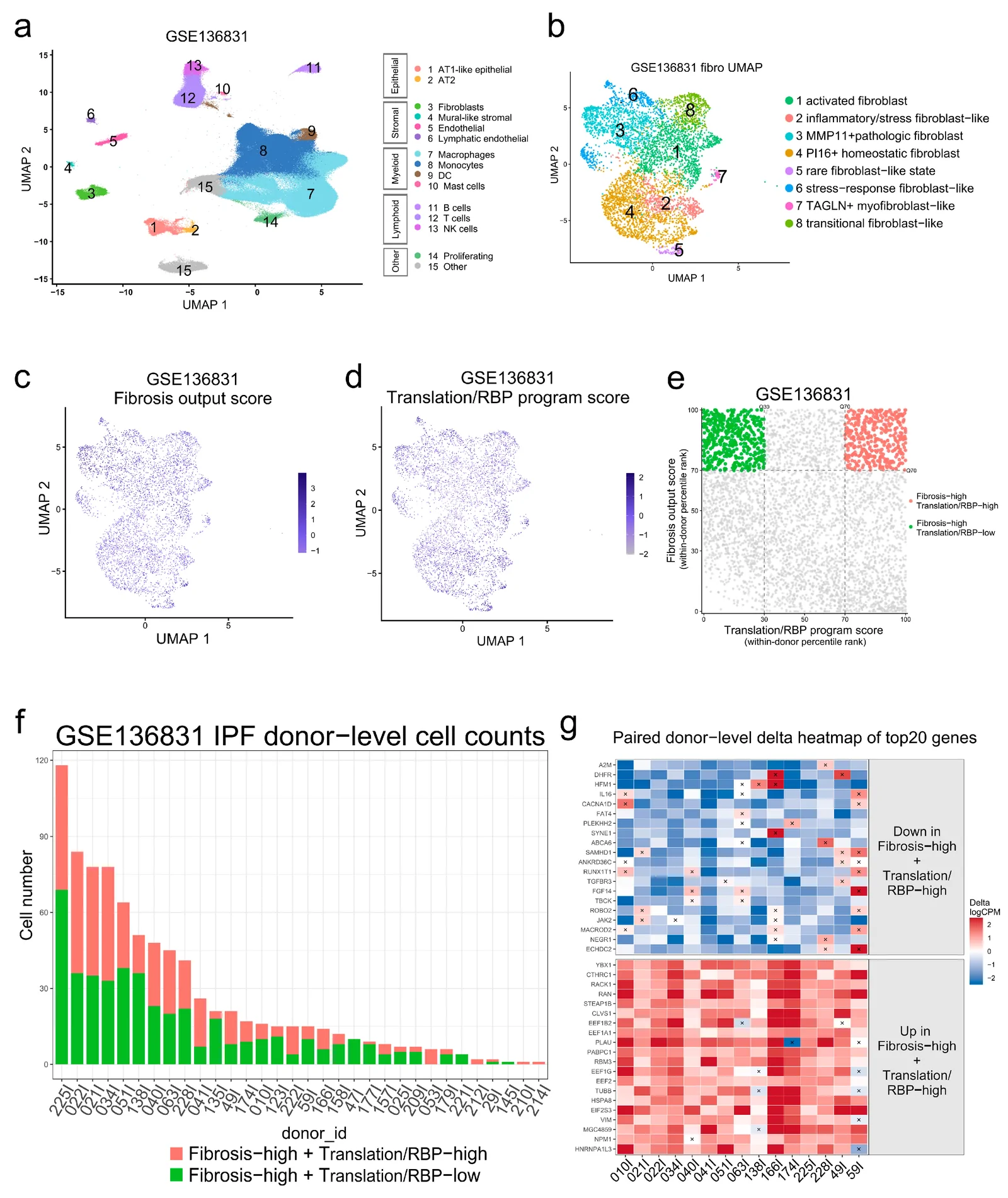
Identification of Translation/RNA-binding protein (RBP)-related transcriptional states and derivation of the discovery signature in GSE136831. (**a**) Uniform Manifold Approximation and Projection (UMAP) representation of 288,909 cells from GSE136831, colored by broad cell-type annotation. (**b**) UMAP representation of 5672 fibroblasts, colored by fibroblast state annotation. (**c**,**d**) UMAP feature plots showing the distributions of the fibrotic output score (**c**) and Translation/RBP program score (**d**) within the fibroblast population. (**e**) Classification of 4566 idiopathic pulmonary fibrosis (IPF) fibroblasts using donor-specific fibrotic output and Translation/RBP program thresholds. Red indicates fibrosis-high/Translation/RBP-high cells; green indicates fibrosis-high/Translation/RBP-low cells; and gray indicates other fibroblasts. (**f**) Numbers of cells assigned to the two target states across 25 IPF donors. Fifteen donors with at least five cells in each state were retained for paired pseudobulk analysis. (**g**) Heatmap of donor-level expression differences for the 20 top-ranked upregulated and 20 top-ranked downregulated genes across the 15 paired donors. Values represent the difference in log_2_ counts per million (logCPM) between the fibrosis-high/Translation/RBP-high and fibrosis-high/Translation/RBP-low states. Crosses (×) indicate donor–gene pairs with a direction opposite to the overall direction for that gene.

Analysis of GSE136831 yielded a fibroblast compartment comprising 5672 cells, including 4566 from IPF lungs (Figure 1a). These fibroblasts were distributed across multiple transcriptionally distinct states (Figure 1b), revealing substantial heterogeneity within the fibroblast population.

We then asked whether this heterogeneity extended to Translation/RBP-related transcriptional variation. Within each IPF donor, fibroblasts with fibrotic output scores at or above the 70th percentile were classified according to their Translation/RBP program scores: cells at or above the donor-specific 70th percentile were defined as fibrosis-high/Translation/RBP-high, whereas those at or below the 30th percentile were defined as fibrosis-high/Translation/RBP-low (Figure 1c–e). Both states were observed across multiple IPF donors, and 15 donors contained sufficient numbers of cells in each state for paired comparison (Figure 1f).

Paired pseudobulk analysis of these 15 donors identified 758 differentially expressed genes, including 489 genes upregulated and 269 downregulated in the fibrosis-high/Translation/RBP-high state, using thresholds of false discovery rate (FDR) < 0.05 and absolute log_2_ fold change (|log_2_FC|) ≥ 0.5. After gene-symbol annotation and removal of mitochondrial, ribosomal, unmapped, and uncharacterized LOC-like features, candidates were further filtered for donor-level directional consistency and to exclude gene symbols unsuitable for downstream connectivity queries, leaving 279 upregulated and 84 downregulated genes. Because the remaining candidate set was strongly skewed toward the upregulated direction, a balanced bidirectional discovery signature was constructed by retaining all 84 downregulated genes and selecting the top 84 upregulated genes based on FDR, effect size, and donor-level directional consistency. This yielded an UP84/DOWN84 discovery signature for subsequent independent directional assessment in GSE135893 (Table S1). Representative genes showed consistent changes across most paired donors (Figure 1g), indicating that the signature captured a donor-consistent transcriptional difference between the two Translation/RBP-related fibroblast states.

### 2.2. Independent Directional Assessment Supports the Cross-Cohort Translation/RBP-Related Signature

**Figure 2:**
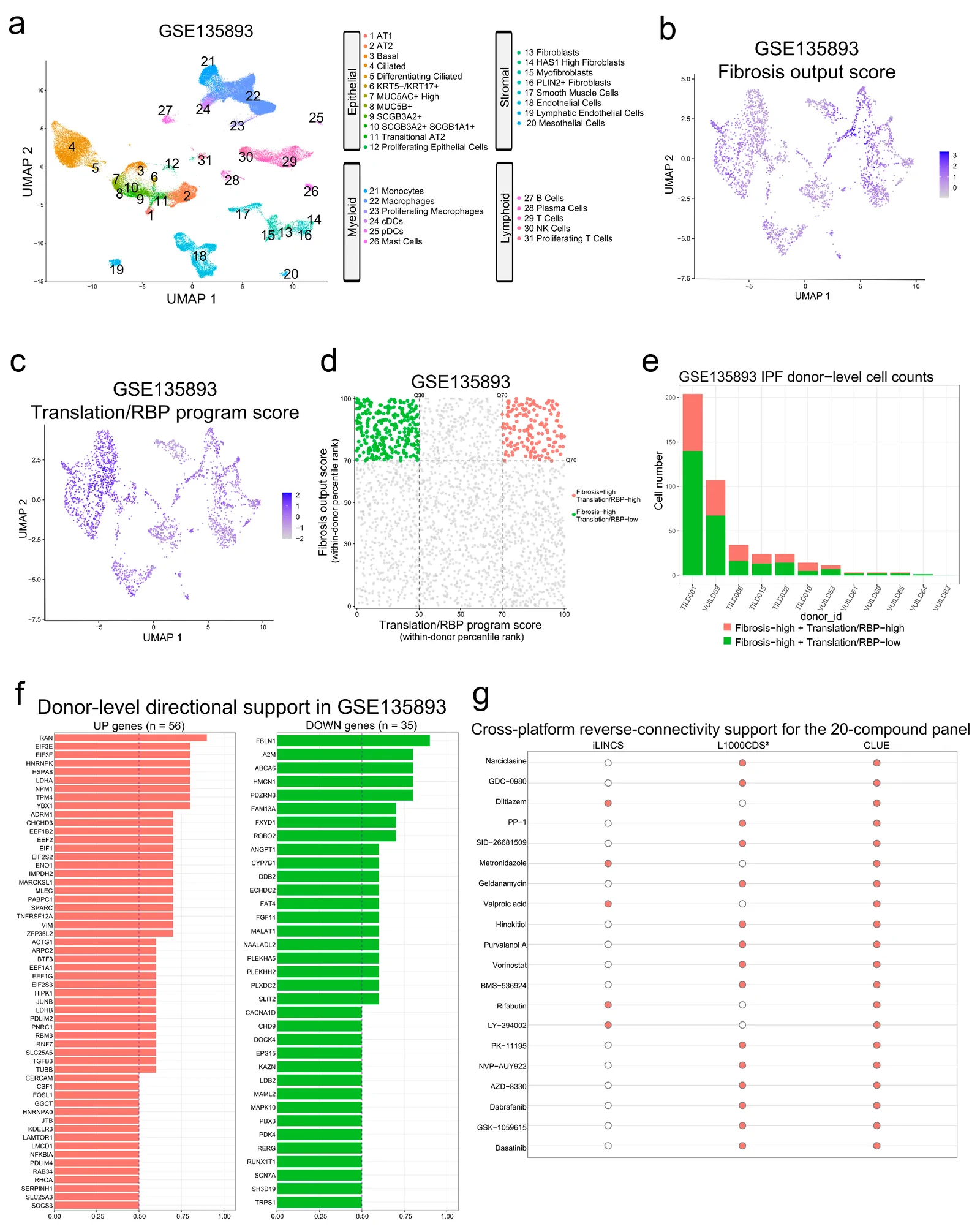
Independent cohort assessment of the transcriptional signature and multi-platform reverse-connectivity analysis. (**a**) Uniform Manifold Approximation and Projection (UMAP) representation of the full GSE135893 dataset comprising 114,396 cells, colored by cell-type annotation. (**b**,**c**) UMAP feature plots showing the distributions of the fibrotic output score (**b**) and Translation/RNA-binding protein (RBP) program score (**c**) within the fibroblast population. (**d**) Classification of 2311 idiopathic pulmonary fibrosis (IPF) fibroblasts using donor-specific fibrotic output and Translation/RBP program thresholds. Red indicates fibrosis-high/Translation/RBP-high cells; green indicates fibrosis-high/Translation/RBP-low cells; and gray indicates other fibroblasts. (**e**) Numbers of cells assigned to the two target states across 12 IPF donors. Ten donors containing both states were retained for independent cohort assessment. (**f**) Proportions of donors showing the discovery-cohort direction for the 56 upregulated (UP) and 35 downregulated (DOWN) genes retained in the cross-cohort signature. The dashed line indicates the 0.50 directional-support threshold. (**g**) Cross-platform support for the 20-compound experimental panel derived from reverse-connectivity analysis using CLUE, L1000CDS^2^, and iLINCS. Filled and open circles indicate the presence and absence of support from the corresponding platform, respectively.

The Translation/RBP-related transcriptional states identified in the discovery cohort were next examined in the independent GSE135893 dataset. Among 2653 fibroblasts, including 2311 from IPF lungs, application of the same state-definition framework recovered both fibrosis-high/Translation/RBP-high and fibrosis-high/Translation/RBP-low populations (Figure 2a–d). Both states were present in 10 IPF donors, enabling paired evaluation of the discovery signature in an independent cohort (Figure 2e).

Independent directional assessment was performed using the predefined UP84/DOWN84 signature in GSE135893. For each gene, the direction of the expression difference between the two states was assessed across the 10 paired donors using trimmed mean of M-values (TMM)-normalized expression. Genes retaining the expected direction in at least 50% of paired donors were considered directionally supported in the independent cohort. This analysis retained 56 UP and 35 DOWN genes, yielding a cross-cohort signature of 91 genes (Figure 2f; Table S2).

To further place the cross-cohort signature in the context of existing IPF biology, we assessed the literature evidence and network positions of its constituent genes. GenCLiP3-assisted text mining and gene-level PubMed review showed that 84.6% of the signature genes had reported associations with IPF, lung fibrosis, or fibroblast/myofibroblast biology, whereas 15.4% remained relatively understudied across these domains (Supplementary Figure S1). In an IPF-related STRING network constructed using DisGeNET-associated genes, many signature genes were either directly annotated to IPF or embedded within the surrounding interaction network, and several occupied relatively central topological positions. Five signature genes ranked within the top 10% of the complete network by degree, two by maximal clique centrality (MCC), and twelve by betweenness, with *CSF1*, *NFKBIA*, *RHOA*, *NPM1*, and *HSPA8* among the representative highly connected genes (Supplementary Figure S2). These analyses place the cross-cohort signature within established IPF- and fibroblast-related biological contexts while also identifying comparatively understudied genes for further investigation.

### 2.3. Multi-Platform Connectivity Analysis Prioritizes Candidate Compounds

The UP56/DOWN35 cross-cohort signature was queried against CLUE, L1000CDS^2^, and iLINCS to identify compounds with opposing transcriptional perturbation profiles. Integration of the three platform-specific results generated a consensus ranking, which was further considered together with compound traceability, reagent availability, and experimental feasibility to define a 20-compound panel for experimental testing (Figure 2g; Supplementary Table S3).

Among the prioritized compounds were valproic acid and vorinostat, both of which have been extensively investigated for their antifibrotic effects in pulmonary fibrosis [22,23,24,25]. Their recovery provided additional support for the disease relevance of the signature-based screen. Given the substantial existing evidence for these compounds, they were retained as literature-supported reference compounds in the initial phenotypic screen, while subsequent evaluation focused on the other prioritized candidates.

### 2.4. Cell Viability Assessment of Candidate Compounds

After valproic acid and vorinostat had been designated as reference compounds, the effects of the remaining 18 candidate compounds on HFL-1 cell viability were evaluated across predefined concentration ranges using the Cell Counting Kit-8 (CCK-8) assay. The complete concentration-dependent viability data are provided in Supplementary Table S4, while Table 1 summarizes the tested concentration range, the highest tested concentration at which mean cell viability remained ≥85%, the corresponding viability value, and the concentration selected for subsequent phenotypic evaluation. The compounds showed distinct tolerance profiles, with the concentration range associated with preserved viability varying substantially among candidates.

Cellular tolerance at the selected concentrations was further assessed by Live/Dead staining and phase-contrast imaging (Supplementary Figure S3). For most compounds, the CCK-8 results were consistent with preserved cell density and morphology. Geldanamycin and NVP-AUY922 were exceptions: at 0.025 μM, mean cell viability remained above 85% (91.6 ± 7.2% and 92.2 ± 2.5%, respectively), but clear morphological alterations were evident in the imaging analyses. These two compounds were therefore not advanced to subsequent antifibrotic phenotypic screening. For the remaining candidates, the CCK-8, Live/Dead, and phase-contrast results were considered together when selecting concentrations for further evaluation.

### 2.5. Phenotypic Screening and Concentration-Response Evaluation of Candidate Compounds

**Figure 3:**
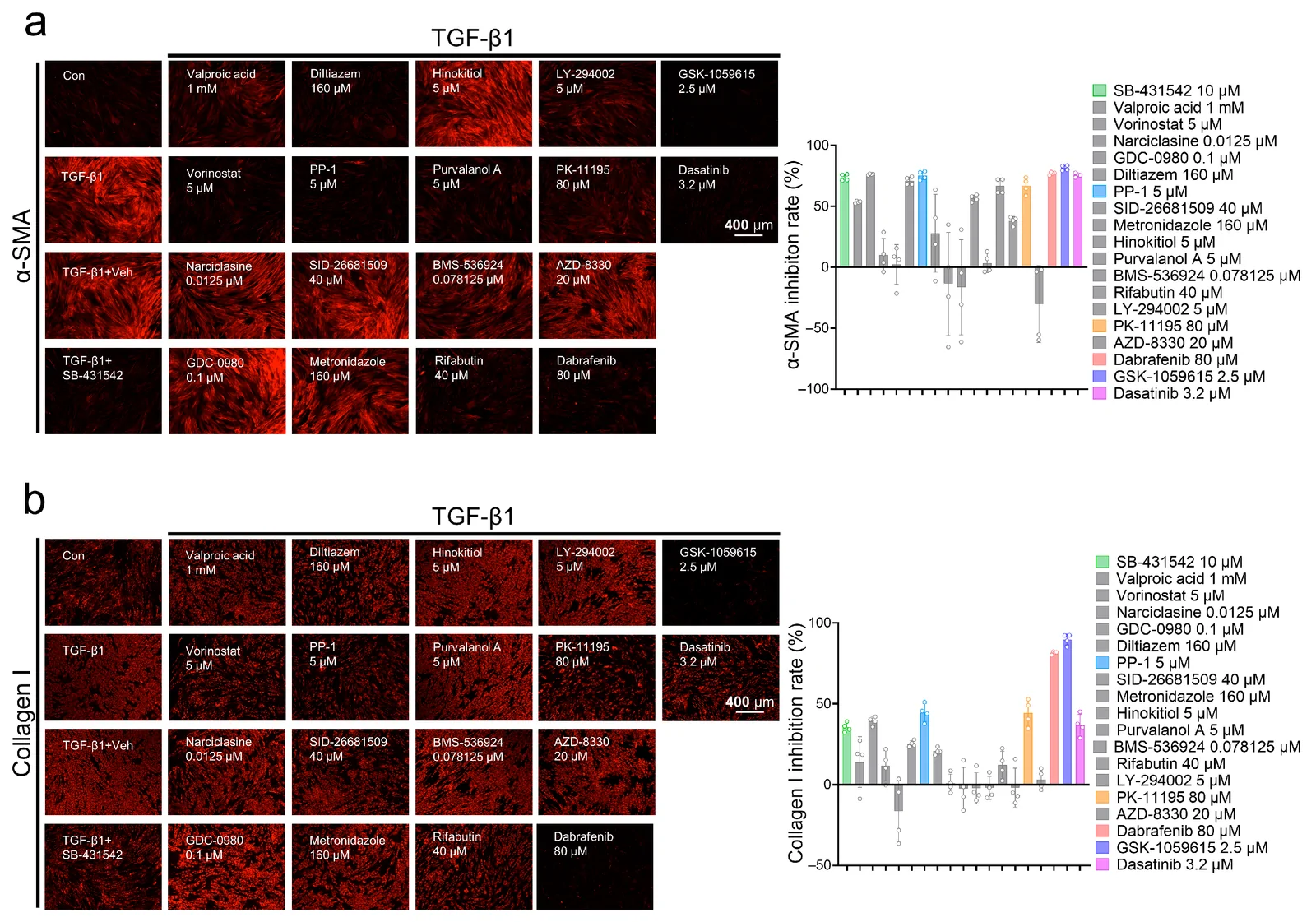
Phenotypic screening of candidate compounds for effects on α-smooth muscle actin (α-SMA) and collagen I expression in transforming growth factor-β1 (TGF-β1)-stimulated human fetal lung fibroblast (HFL-1) cells. (**a**) Representative α-SMA immunofluorescence images and corresponding quantification following treatment with the indicated compounds. (**b**) Representative collagen I immunofluorescence images and corresponding quantification. Valproic acid and vorinostat were included as literature-supported reference compounds, and SB-431542 was used as the pharmacological positive control. Con, unstimulated control; TGF-β1, TGF-β1-stimulated cells without additional treatment; Veh, TGF-β1-stimulated cells treated with vehicle. Inhibition was calculated relative to the TGF-β1 + vehicle group, which was defined as 0% inhibition; positive values indicate lower fluorescence intensity than the vehicle group, whereas negative values indicate higher fluorescence intensity. Data are presented as mean ± standard deviation (SD) (*n* = 4). Scale bars, 400 μm.

The candidate compounds that passed the preceding cellular tolerance assessment were next evaluated in TGF-β1-stimulated HFL-1 cells, together with valproic acid and vorinostat as literature-supported reference compounds. TGF-β1 markedly increased α-smooth muscle actin (α-SMA) and collagen I expression compared with unstimulated controls, whereas SB-431542 substantially reduced both markers, confirming the expected responsiveness of the assay (Figure 3a,b). Valproic acid and vorinostat also reduced α-SMA and collagen I expression. The candidate compounds showed heterogeneous effects on the two profibrotic markers. PP-1, PK-11195, dabrafenib, GSK-1059615, and dasatinib produced concordant reductions in both α-SMA and collagen I and were selected for further concentration-response evaluation. Other compounds showed weaker or more selective effects on one of the two markers.

**Figure 4:**
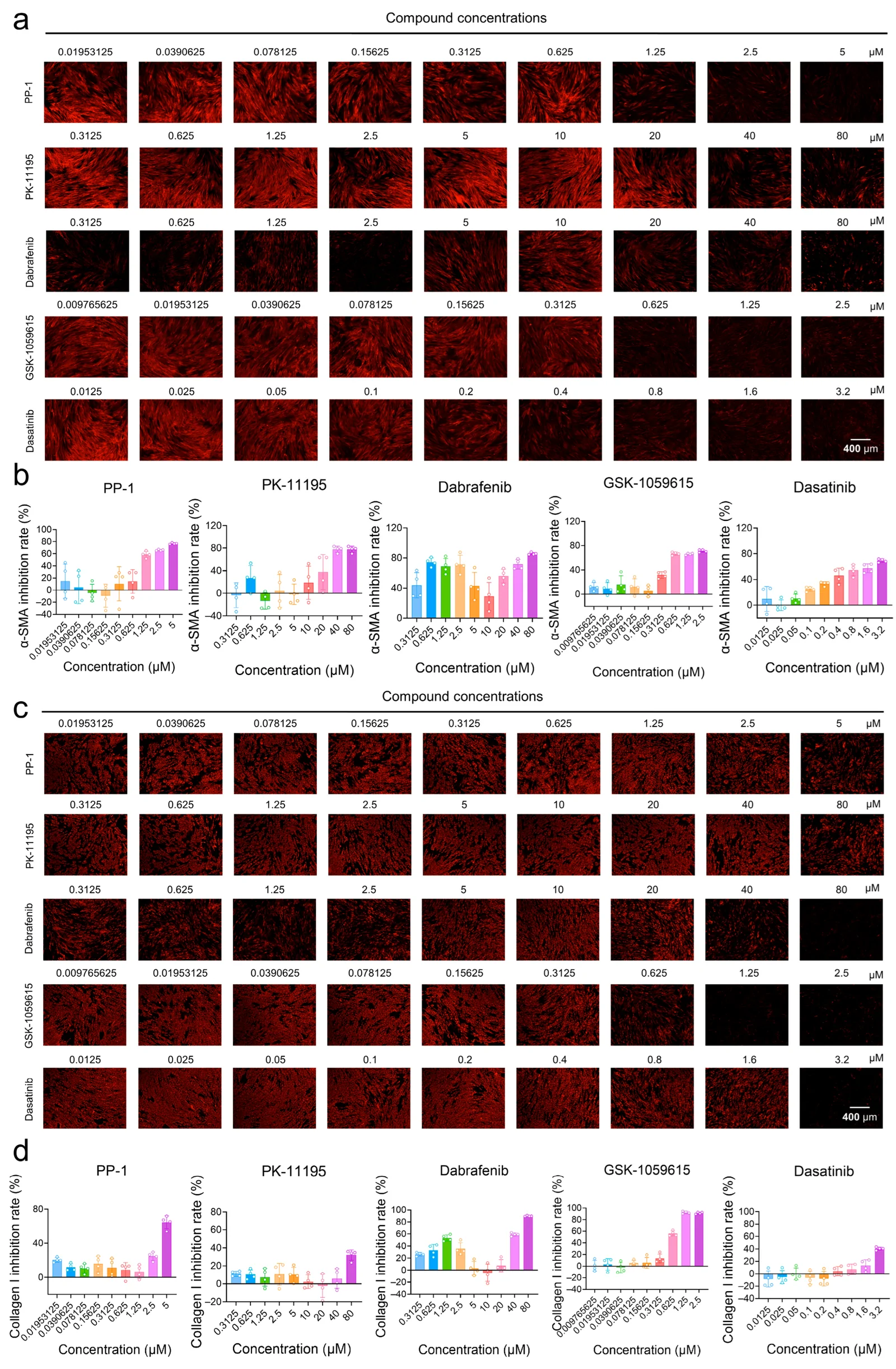
Concentration–response effects of five candidate compounds on α-smooth muscle actin (α-SMA) and collagen I expression in transforming growth factor-β1 (TGF-β1)-stimulated human fetal lung fibroblast (HFL-1) cells. (**a**) Representative α-SMA immunofluorescence images of cells treated with the indicated concentrations of PP-1, PK-11195, dabrafenib, GSK-1059615, and dasatinib. (**b**) Quantification of α-SMA inhibition across the tested concentration ranges. (**c**) Representative collagen I immunofluorescence images following treatment with the same compounds. (**d**) Quantification of collagen I inhibition across the tested concentration ranges. Inhibition was calculated relative to the TGF-β1 + vehicle group, which was defined as 0% inhibition; positive values indicate lower fluorescence intensity than the vehicle group, whereas negative values indicate higher fluorescence intensity. Data are presented as mean ± standard deviation (SD) (*n* = 4). Scale bars, 400 μm.

The five selected compounds were subsequently examined across a broader concentration range by immunofluorescence (Figure 4). Distinct concentration–response patterns were observed for the two markers. PP-1 produced little inhibition of α-SMA at lower concentrations, followed by a marked increase in activity from 1.25 to 5 μM; its effect on collagen I was comparatively modest over most of the range and became pronounced mainly at 5 μM. PK-11195 similarly showed limited activity at lower concentrations, with α-SMA inhibition increasing substantially at 20–80 μM and the strongest collagen I inhibition observed at 80 μM. Dabrafenib displayed a non-monotonic profile: both markers were reduced at low concentrations, the effect weakened at intermediate concentrations, and inhibition increased again at 40–80 μM, particularly for collagen I. GSK-1059615 showed a clearer concentration-related response, with inhibition of both α-SMA and collagen I rising sharply at the upper end of the tested range. Dasatinib also showed progressively greater inhibition of α-SMA with increasing concentration, whereas its effect on collagen I was weaker and became evident mainly at the higher concentrations.

**Figure 5:**
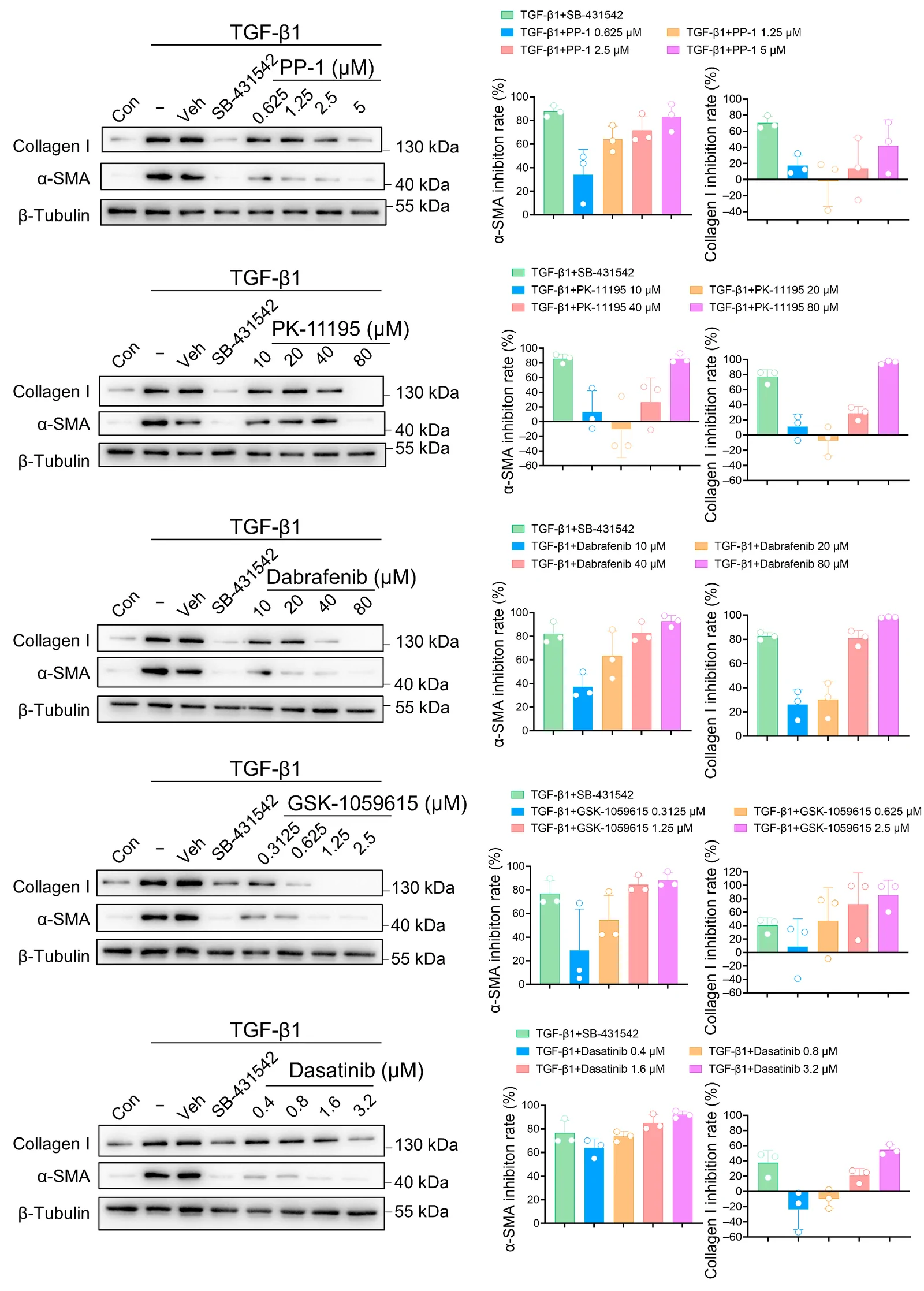
Western blot assessment of the effects of five candidate compounds on α-smooth muscle actin (α-SMA) and collagen I expression in transforming growth factor-β1 (TGF-β1)-stimulated human fetal lung fibroblast (HFL-1) cells. Representative Western blots and corresponding quantification of α-SMA and collagen I expression following treatment with PP-1, PK-11195, dabrafenib, GSK-1059615, or dasatinib at the indicated concentrations. SB-431542 was used as the pharmacological positive control. β-Tubulin was used as the loading control. Protein levels were normalized to β-tubulin, and inhibition was calculated relative to the TGF-β1 + vehicle group, which was defined as 0% inhibition. Positive values indicate lower protein expression than the vehicle group, whereas negative values indicate higher expression. Data are presented as mean ± standard deviation (SD) (*n* = 3).

Western blotting was then used as a complementary assay to further verify the effects of these five compounds (Figure 5). The results generally supported the patterns observed by immunofluorescence, although the magnitude of the responses differed between the two assays. PP-1 progressively reduced α-SMA across the tested concentrations, while its effect on collagen I was less uniform and was strongest at 5 μM. PK-11195 showed little effect at 10–20 μM but markedly reduced both markers at 80 μM. Dabrafenib produced progressively stronger reductions in both α-SMA and collagen I from 10 to 80 μM, with prominent inhibition at 40–80 μM. GSK-1059615 reduced both markers across the tested range, with stronger effects at 0.625–2.5 μM. Dasatinib consistently suppressed α-SMA, whereas substantial reduction in collagen I was observed mainly at 1.6–3.2 μM. Together, the immunofluorescence and Western blot results showed that the five compounds differed not only in the concentrations at which their effects emerged but also in the relative extent to which they affected α-SMA and collagen I.

## 3. Discussion

This study identified Translation/RBP-related transcriptional states within the fibroblast compartment of human IPF lungs and derived a cross-cohort signature from the expression differences between these states. Directional consistency across donors and independent directional assessment in a second cohort yielded a 91-gene signature that was subsequently used for reverse-connectivity analysis. Compounds prioritized through this approach were experimentally evaluated in TGF-β1-stimulated HFL-1 fibroblasts, linking a fibroblast state-associated transcriptional signature to compounds with measurable effects on profibrotic phenotypic readouts.

Previous studies have implicated translational control and individual RBPs in fibroblast activation and pulmonary fibrosis [8,9,10,11,12]. Extending these observations to the heterogeneity of human IPF fibroblasts, we identified Translation/RBP-related transcriptional states and derived a cross-cohort transcriptional signature from the differences between them. Beyond its use in reverse-connectivity analysis, this signature could be applied to additional single-cell and spatial transcriptomic datasets to determine how the corresponding state is distributed across patients and whether it is preferentially associated with particular fibroblast populations, fibrotic lesions, or disease-associated tissue niches. This question is particularly relevant given recent spatial transcriptomic studies showing that fibroblast states occupy distinct molecular and anatomical niches in pulmonary fibrosis [26,27]. The same signature could also be used to assess molecular responses to compound treatment. Transcriptomic profiling after treatment would allow direct testing of whether compounds prioritized by reverse connectivity shift the Translation/RBP-related expression pattern in the predicted direction [28]. Combined with measurements of α-SMA and collagen I, such analyses would determine whether suppression of fibroblast activation and collagen production is accompanied by reversal of the molecular state used for compound prioritization and could help distinguish compounds acting through this program from those that reduce fibrotic output through other mechanisms. Finally, integration of the signature with gene-regulatory network analysis could help identify upstream regulators associated with the Translation/RBP-related state difference. Regulon-based analyses of single-cell transcriptomes have been used to link cell-state signatures to upstream regulatory programs [29] and could therefore be applied here to prioritize candidate regulators for experimental validation.

Cell-based phenotypic evaluation provided an experimental assessment of the compounds prioritized from the Translation/RBP-related signature. Valproic acid and vorinostat, both of which have prior experimental evidence of antifibrotic activity in pulmonary fibrosis [22,23,24,25], reduced α-SMA and collagen I expression in the present model, and several additional candidates showed similar effects. Importantly, neither α-SMA nor collagen I was included in the transcriptional signature used for compound prioritization. The observed effects on these markers therefore provide independent experimental evidence that compounds identified from the Translation/RBP-related signature can modulate fibroblast activation and collagen production. Whether these effects are accompanied by reversal of the Translation/RBP-related transcriptional state remains to be determined.

PP-1, PK-11195, dabrafenib, GSK-1059615, and dasatinib differ substantially in their pharmacological backgrounds and in the extent to which their activity can be related to established fibrotic mechanisms. PP-1 and dasatinib both target Src-family kinase signaling, which has been implicated in fibroblast activation and pulmonary fibrosis. Pharmacological inhibition of Src has been shown to suppress TGF-β1-induced myofibroblast differentiation and extracellular matrix production in human lung fibroblasts and to attenuate bleomycin-induced pulmonary fibrosis in mice [30]. Dasatinib has likewise been reported to suppress TGF-β-induced myofibroblast differentiation in fibrotic human lung fibroblasts through Src–serum response factor (SRF) signaling and to attenuate bleomycin-induced pulmonary fibrosis in mice [31,32]. These findings are consistent with the reductions in α-SMA and collagen I observed in our HFL-1 model. However, the previously reported effects of dasatinib on human lung fibroblast activation occurred at nanomolar concentrations, whereas pronounced collagen I suppression in our experiments was observed mainly at 1.6–3.2 μM, indicating that the concentration dependence of the response differs between experimental systems. GSK-1059615 is a dual phosphoinositide 3-kinase (PI3K)/mechanistic target of rapamycin (mTOR) inhibitor, providing a mechanistically plausible link to fibroblast activation because aberrant PI3K/mTOR signaling has been implicated in IPF. Although GSK-1059615 itself has not been evaluated in IPF fibroblasts, pharmacological PI3K/mTOR inhibition with GSK2126458 suppressed functional responses in IPF-derived lung fibroblasts and inhibited PI3K signaling in human IPF lung tissue [33]. Dabrafenib has previously been identified as a candidate for IPF drug repurposing [34], and experimental studies also support its activity in fibrotic settings. In human cardiac fibroblasts, dabrafenib inhibited extracellular signal-regulated kinase 1/2 (ERK1/2) signaling at approximately 10 μM, and in vivo treatment reduced cardiac fibrosis in a murine model of angiotensin II-induced hypertension [35]. Dabrafenib has also been shown to attenuate experimental renal fibrosis through pharmacological inhibition of receptor-interacting protein kinase 3 (RIPK3) [36]. In our HFL-1 model, dabrafenib showed a non-monotonic concentration–response pattern by immunofluorescence, whereas the strongest and most consistent reductions in α-SMA and collagen I were observed at 40–80 μM. Thus, although the antifibrotic phenotype is consistent with previous observations in other fibroblast and fibrosis models, the concentrations associated with the strongest effects in our assay were higher than those used in previous cellular studies. They also exceed the systemic plasma concentrations reported with the standard clinical dose of dabrafenib (150 mg twice daily) [37,38]. Together with the non-monotonic response observed in our assay, these findings suggest that the effects of dabrafenib in HFL-1 cells cannot be explained simply by a conventional concentration-dependent response to a single pharmacological target. PK-11195 is a high-affinity ligand of the mitochondrial translocator protein (TSPO), with reported TSPO binding affinities in the low-nanomolar range [39]. In contrast, the strongest effects on α-SMA and collagen I in our experiments occurred at 80 μM. At micromolar concentrations, PK-11195 has been reported to produce biological effects that are not necessarily mediated by TSPO, including mitochondrial effects and TSPO-independent regulation of steroidogenesis [40,41]. Thus, the activity of PK-11195 observed here cannot presently be attributed specifically to TSPO.

Several limitations should be considered. The Translation/RBP program was inferred from transcriptomic data and therefore does not directly measure translational activity, RNA stability, or the activity of individual RBPs. The present analysis defines an expression state associated with Translation/RBP-related processes without establishing the causal mechanisms that generate or maintain this state. The two target states were operationally defined using donor-specific score thresholds and should therefore be interpreted as contrasting expression states; this classification does not establish discrete fibroblast subtypes. Furthermore, the perturbational signatures queried across CLUE, L1000CDS^2^, and iLINCS were generated in diverse cellular contexts, which may limit their direct transferability to IPF fibroblasts. The pharmacological experiments were performed in TGF-β1-stimulated HFL-1 cells, which provide a controlled human lung fibroblast model but do not fully capture the donor-specific and disease-associated properties of primary IPF fibroblasts. Validation in patient-derived primary lung fibroblasts, together with direct interrogation of Translation/RBP-related mechanisms and in vivo studies, will be important for assessing the robustness, mechanistic basis, and disease relevance of these findings.

## 4. Materials and Methods

### 4.1. Single-Cell RNA-Seq Datasets and Data Processing

Human lung single-cell RNA-sequencing datasets GSE136831 and GSE135893 were obtained from the Gene Expression Omnibus (GEO) database. GSE136831 was used as the discovery cohort, whereas GSE135893 served as the independent cohort for directional assessment. For donor-level comparisons, each donor was treated as an independent biological unit.

For GSE136831, analysis started from the merged Seurat object and was restricted to cells from Control and IPF samples. Cells with nCount_RNA between 1000 and 30,000 and nFeature_RNA between 200 and 6000 were retained, and samples with fewer than 500 cells after quality control were excluded. RNA counts were normalized using the LogNormalize method with a scale factor of 10,000, and 3000 highly variable genes were identified using the vst method. Dimensional reduction and clustering were then performed using the Seurat (version 5.4.0) sketch-based workflow, including ScaleData, principal component analysis (PCA), neighbor-graph construction, clustering, and Uniform Manifold Approximation and Projection (UMAP). The resulting dimensional-reduction and clustering information was projected back onto the full dataset using ProjectData. Cell identities were confirmed based on available annotation metadata, cluster-level expression profiles, and established marker genes. Fibroblasts were identified primarily according to markers including *SFRP2*, *LUM*, *COL1A1*, *COL1A2*, *COL3A1*, *DCN*, and *CD248*, yielding 5672 fibroblasts.

For GSE135893, analysis was performed using the full single-cell object containing 114,396 cells and 33,694 gene features. RNA expression data were normalized using the LogNormalize method with a scale factor of 10,000, and 3000 highly variable genes were identified using the vst method. Dimensional reduction and clustering were subsequently performed using the Seurat (version 5.4.0) sketch-based workflow, including PCA, neighbor-graph construction, clustering, and UMAP, followed by projection of the resulting information onto the full dataset using ProjectData. Cell identities were assigned using annotation information provided by the original study. Subsequent analyses were restricted to control and IPF samples, comprising 89,326 cells, from which 2653 fibroblasts were obtained.

Downstream analyses were performed within the full fibroblast populations of the respective datasets.

### 4.2. Definition of Translation/RBP-Related Transcriptional States

Fibrotic output and Translation/RBP-related program scores were evaluated separately within the fibroblast compartment. The fibrotic output score was calculated with AddModuleScore using a predefined 10-gene set consisting of *ACTA2*, *COL1A1*, *COL1A2*, *COL3A1*, *FN1*, *POSTN*, *CTHRC1*, *TNC*, *THBS1*, and *LOX*.

The Translation/RBP program comprised four predefined functional modules representing translation initiation, RNA-binding proteins and RNA helicases, stress granule/ribonucleoprotein dynamics, and mRNA fate regulation. Together, these modules contained 59 module–gene records corresponding to 53 unique genes. Ribosomal and mitochondrial ribosomal genes, including RPL/RPS and MRPL/MRPS family members, were excluded from the seed sets. For each module, the normalized expression value of each member gene was first z-standardized across the fibroblast compartment. The resulting gene-level z-scores were then averaged within each module to generate a cell-level raw module score. Each raw module score was subsequently z-standardized across fibroblasts, and the overall Translation/RBP program score was calculated as the equal-weight mean of the four standardized module scores (Table S5).

The Translation/RBP-related transcriptional states were operationally defined by the combined positions of cells along the fibrotic output and Translation/RBP program score distributions within each donor. Target-state assignment was restricted to IPF fibroblasts and performed separately within each donor. The donor-specific 70th percentile of the fibrotic output score and the 30th and 70th percentiles of the Translation/RBP program score were used as thresholds. For visualization of the state-definition framework in Figure 1e and Figure 2d, both scores were expressed as within-donor percentile ranks. Fibroblasts with a fibrotic output score at or above the 70th percentile and a Translation/RBP score at or above the 70th percentile were classified as fibrosis-high/Translation/RBP-high. Fibroblasts meeting the same fibrotic output threshold but with a Translation/RBP score at or below the 30th percentile were classified as fibrosis-high/Translation/RBP-low. Other fibroblasts were not included in the state comparison. For GSE136831, donors were required to contain at least five cells in each target state, yielding 15 paired donors. For GSE135893, donors containing both target states were retained for independent directional assessment, yielding 10 paired donors.

### 4.3. Discovery Signature Construction and Independent Directional Assessment

In the GSE136831 discovery cohort, raw RNA counts from the two target states were aggregated by donor and state across 15 paired donors, generating 30 pseudobulk samples. Differential expression analysis was performed using edgeR (version 4.8.2). Lowly expressed genes were filtered using filterByExpr, followed by library-size normalization using the trimmed mean of M-values (TMM) method. Differential expression was then assessed using a quasi-likelihood generalized linear model with a paired design, in which donor identity was included as a blocking factor and cell state was the variable of interest. log_2_FC was defined as fibrosis-high/Translation/RBP-high relative to fibrosis-high/Translation/RBP-low. *p* values were adjusted using the Benjamini–Hochberg method, and genes with FDR < 0.05 and |log_2_FC| ≥ 0.5 were considered differentially expressed.

For subsequent signature construction, Ensembl identifiers were mapped to gene symbols, and duplicated mappings were resolved before downstream analysis. Mitochondrial genes, ribosomal and mitochondrial ribosomal genes, unmapped features, uncharacterized LOC-like genes, and noncoding or other gene symbols unsuitable for subsequent connectivity analysis were excluded.

Directional consistency of differential expression was further evaluated across the 15 paired donors. Candidate genes for signature construction were required to meet the thresholds of FDR < 0.05 and |log_2_FC| ≥ 0.5 and to show the same direction of change as the overall differential expression result in at least 75% of paired donors. These criteria yielded 279 upregulated and 84 downregulated candidate genes. Because this candidate set was strongly skewed toward the upregulated direction, a balanced bidirectional discovery signature was constructed before assessment in the independent cohort. To construct a numerically balanced discovery signature, the 279 upregulated candidates were ranked according to FDR, |log_2_FC|, and donor-level directional consistency, and the top 84 genes were retained. All 84 downregulated candidates were retained, resulting in an UP84/DOWN84 discovery signature.

The predefined UP84/DOWN84 signature was subsequently subjected to independent directional assessment in the GSE135893 cohort. Of the 168 input genes, 158 could be evaluated in this dataset. Raw RNA counts from the two target states were aggregated by donor and state across the 10 paired donors, generating 20 pseudobulk samples. TMM normalization factors were calculated from the full pseudobulk count matrix using edgeR, and normalized expression values were converted to logCPM. The 158 evaluable signature genes were then extracted for donor-level directional assessment. For each gene, the direction of expression change between the two target states was assessed across 10 paired donors. Genes showing the same direction as in the discovery cohort in at least 50% of donors were considered directionally supported in the independent cohort. No independent differential-expression significance threshold was applied in GSE135893. This analysis yielded a cross-cohort signature comprising 56 UP genes and 35 DOWN genes.

The cross-cohort signature was further characterized by literature and network analyses. GenCLiP3 text mining and gene-level PubMed review were used to assess evidence in IPF, lung fibrosis, and fibroblast/myofibroblast biology. For network analysis, IPF-associated genes from DisGeNET were combined with the signature genes and mapped to a high-confidence STRING (version 12.5) functional interaction network (interaction score ≥ 0.700; no additional interactors), followed by visualization in Cytoscape (version 3.10.5; Cytoscape Consortium, San Diego, CA, USA). Degree, maximal clique centrality (MCC), clustering coefficient, betweenness, and harmonic closeness were calculated across the complete network.

### 4.4. Reverse-Connectivity Analysis

The final UP56/DOWN35 signature was queried separately against CLUE, L1000CDS^2^, and iLINCS to identify compounds whose transcriptional perturbation profiles were inversely associated with the target fibroblast signature.

For CLUE, records were retained if they represented compound treatments (pert_type = trt_cp), had TAG < 0, satisfied is_ncs_sig = 1, and satisfied either qc_pass = 1 or is_hiq = 1. When multiple records were available for the same compound, the record with the most negative TAG was retained. For L1000CDS^2^, a reverse query was performed, and results with Rank ≤ 50 and score > 0 were retained. When multiple records were available for the same compound, the result with the lowest rank was retained, with the higher score used in the case of tied ranks. For iLINCS, results with Concordance < 0 and *p* < 0.05 were retained, and the result with the most negative Concordance was retained for compounds with multiple records, with the lower *p* value used in the case of tied Concordance values.

Compound names were standardized across platforms before integration. Compounds within each platform were ranked according to the corresponding reverse-connectivity evidence, and the ranks were converted to normalized rank scores using Srank = 1 − (r − 1)/(N − 1), where r denotes the rank of a compound within a given platform, and N denotes the total number of compounds included in that platform-specific ranking. The cross-platform consensus ranking was determined sequentially according to the number of supporting platforms, the mean normalized rank score across supporting platforms, and the best normalized rank score.

Following completion of the consensus ranking, the compound panel used for subsequent experimental evaluation comprised 20 compounds, with its composition determined by considering the consensus ranking together with compound traceability, reagent availability, and experimental feasibility.

### 4.5. HFL-1 Cell Culture and TGF-β1 Treatment

Human fetal lung fibroblasts (HFL-1, CL-0106, Procell, Wuhan, China) were cultured in HFL-1 complete medium (CM-0106, Procell) at 37 °C with 5% CO_2_. For antifibrotic experiments, cells were seeded in 96-well plates at 2.0 × 10^4^ cells/well for immunofluorescence analysis or in 12-well plates at 2.0 × 10^5^ cells/well for Western blot analysis and allowed to reach approximately 70–80% confluence. The culture medium was then replaced with serum-free F-12K medium (iCell-0007, iCell, Shanghai, China), and the cells were serum-starved for 24 h. After serum starvation, cells were pretreated with candidate compounds for 1 h before stimulation with recombinant human TGF-β1 (CA59, Novoprotein, Suzhou, China) at a final concentration of 10 ng/mL for 36 h.

### 4.6. Cell Viability and Live/Dead Assays

Cell viability and tolerance of HFL-1 cells to candidate compounds were evaluated using CCK-8 (GK10001, GlpBio, Montclair, CA, USA) and a Calcein-AM/propidium iodide (PI) Live/Dead staining kit (BL130A, Biosharp, Beijing, China). Cells were treated with candidate compounds at different concentrations for 36 h.

The CCK-8 assay was performed according to the manufacturer’s instructions. After compound treatment, CCK-8 working solution was added, and the cells were incubated at 37 °C for 1 h. Absorbance at 450 nm was measured using an Infinite M Plex multimode microplate reader (Tecan, Männedorf, Switzerland). After subtraction of the blank signal, relative cell viability was calculated using the untreated control as the reference.

Live/Dead staining with Calcein-AM and PI was performed according to the manufacturer’s instructions. Following staining, cells were imaged using the Incucyte S3 Live-Cell Analysis System (Sartorius, Göttingen, Germany).

### 4.7. Immunofluorescence Staining and Image Acquisition

After treatment, cells were fixed with 4% paraformaldehyde (G1101, Servicebio, Wuhan, China) for 20 min at room temperature, permeabilized with 0.1% Triton X-100 (V900502, Sigma-Aldrich, St. Louis, MO, USA) for 15 min, and blocked with 5% normal goat serum (SL038, Solarbio, Beijing, China) containing 1% bovine serum albumin (BSA; A850221, Macklin, Shanghai, China) for 30 min. Cells were incubated overnight at 4 °C with either an anti-α-SMA primary antibody (Sigma-Aldrich, St. Louis, MO, USA; A2547; 1:400) or an anti-collagen I primary antibody (Abiowell, Changsha, China; AWA10926; 1:200). Corresponding Alexa Fluor 594 goat anti-mouse (CST, Danvers, MA, USA; 8890S; 1:2000) or goat anti-rabbit (CST, Danvers, MA, USA; 8889S; 1:2000) secondary antibodies were then applied for 1 h at room temperature in the dark. Immunofluorescence images were acquired using the Incucyte S3 Live-Cell Analysis System (Sartorius, Göttingen, Germany).

### 4.8. Western Blot Analysis

HFL-1 cells were treated with the indicated compounds under the experimental conditions described above and lysed using RIPA buffer (P0013B, Beyotime, Shanghai, China) supplemented with cOmplete protease inhibitor cocktail (04693116001, Roche Diagnostics, Mannheim, Germany) and PhosSTOP phosphatase inhibitor cocktail (04906837001, Roche Diagnostics, Mannheim, Germany). Protein concentrations were determined using a bicinchoninic acid (BCA) protein assay kit (Vazyme, E112-02, Nanjing, China). Equal amounts of protein were separated by SDS-PAGE and transferred onto polyvinylidene difluoride (PVDF) membranes (Immobilon-P, IPVH00010, Merck Millipore, Darmstadt, Germany). After blocking with 5% non-fat milk for 1 h at room temperature, membranes were incubated with primary antibodies against α-SMA (Abiowell, Changsha, China; AWA02626, 1:2500), collagen I (Abiowell, Changsha, China; AWA10926, 1:2500), and β-tubulin (Proteintech, Wuhan, China; 10094-1-AP, 1:10,000) at 4 °C overnight. Following incubation with the appropriate horseradish peroxidase (HRP)-conjugated secondary antibodies, including goat anti-rabbit IgG (7074S, Cell Signaling Technology, Danvers, MA, USA) and horse anti-mouse IgG (7076S, Cell Signaling Technology, Danvers, MA, USA), protein signals were detected using an enhanced chemiluminescence (ECL) detection reagent (GenSuper ECL, JXE0011, GenXion, Guangzhou, China). Band intensities were quantified using ImageJ software (version 1.54g; National Institutes of Health, Bethesda, MD, USA) and normalized to the corresponding loading control.

### 4.9. Statistical Analysis

Immunofluorescence and Western blot signals were quantified using ImageJ (version 1.54g; National Institutes of Health, Bethesda, MD, USA). For both assays, inhibition was expressed relative to the TGF-β1 + vehicle group, which was set to 0%; negative values therefore indicate expression above the vehicle reference. Cell-based experimental data were analyzed and plotted using GraphPad Prism (version 9.5; GraphPad Software, Boston, MA, USA). Single-cell transcriptomic analyses were performed in R (version 4.5.2; R Foundation for Statistical Computing, Vienna, Austria). For analyses involving multiple hypothesis testing, *p* values were adjusted using the Benjamini–Hochberg method, with FDR < 0.05 considered statistically significant.

## 5. Conclusions

This study identified Translation/RBP-related transcriptional states in fibroblasts from human IPF lungs and derived a cross-cohort signature from their expression differences. Reverse-connectivity analysis of this signature across multiple perturbational platforms prioritized compounds with predicted opposing transcriptional effects, several of which subsequently reduced TGF-β1-induced α-SMA and collagen I expression in HFL-1 cells. These findings support the use of fibroblast state-derived transcriptional signatures to prioritize experimentally testable candidates for drug repurposing in IPF.

## Figures and Tables

**Table 1:** Summary of HFL-1 cell viability and concentrations selected for phenotypic screening.

Compound	Tested Range (μM)	Highest Concentration with Mean Viability ≥ 85% (μM)	Mean Viability at That Concentration (%)	Screening Concentration (μM)
Narciclasine	0–3.2	0.0125	85.1 ± 3.6	0.0125
GDC-0980	0–3.2	0.1	85.1 ± 2.9	0.1
Geldanamycin	0–3.2	0.025	91.6 ± 7.2	— ^a^
NVP-AUY922	0–3.2	0.025	92.2 ± 2.5	— ^a^
Dasatinib	0–3.2	3.2	100.6 ± 3.7	3.2
PP-1	0–20	5	91.0 ± 6.8	5
Hinokitiol	0–20	5	89.5 ± 2.6	5
Purvalanol A	0–20	5	101.9 ± 16.2	5
BMS-536924	0–20	0.078125	100.0 ± 10.6	0.078125
LY-294002	0–20	5	93.8 ± 3.8	5
AZD-8330	0–20	20	91.6 ± 5.7	20
GSK-1059615	0–20	2.5	89.8 ± 1.9	2.5
Diltiazem	0–160	160	89.6 ± 2.0	160
SID-26681509	0–160	40	96.8 ± 4.6	40
Metronidazole	0–160	160	98.1 ± 4.5	160
Rifabutin	0–160	40	87.0 ± 3.5	40
PK-11195	0–160	80	102.5 ± 2.8	80
Dabrafenib	0–160	80	91.9 ± 4.0	80

Values are presented as mean ± standard deviation (SD) (*n* = 6) and normalized to the untreated control (100%). Before phenotypic evaluation, concentrations were selected to ensure that mean cell viability remained at or above 85%, with Live/Dead staining and phase-contrast morphology also taken into consideration. ^a^ Although mean cell viability remained ≥85%, geldanamycin and NVP-AUY922 were not advanced to phenotypic screening because marked morphological alterations were observed by Live/Dead staining and phase-contrast imaging (Supplementary Figure S3).

## Data Availability

The original contributions presented in this study are included in the article and Supplementary Materials. The single-cell RNA-sequencing datasets analyzed in this study are publicly available in the Gene Expression Omnibus (GEO) under accession numbers GSE136831 and GSE135893. Further inquiries can be directed to the corresponding author.

## Supplementary Materials

The following supporting information can be downloaded at https://www.mdpi.com/article/10.3390/ijms27219736/s1.

## References

[1] Henderson N.C., Rieder F., Wynn T.A. (2020). Fibrosis: From mechanisms to medicines. Nature.

[2] Lederer D.J., Martinez F.J. (2018). Idiopathic Pulmonary Fibrosis. N. Engl. J. Med..

[3] Wolters P.J., Collard H.R., Jones K.D. (2014). Pathogenesis of idiopathic pulmonary fibrosis. Annu. Rev. Pathol..

[4] Tsukui T., Sun K.H., Wetter J.B., Wilson-Kanamori J.R., Hazelwood L.A., Henderson N.C., Adams T.S., Schupp J.C., Poli S.D., Rosas I.O. (2020). Collagen-producing lung cell atlas identifies multiple subsets with distinct localization and relevance to fibrosis. Nat. Commun..

[5] King T.E., Bradford W.Z., Castro-Bernardini S., Fagan E.A., Glaspole I., Glassberg M.K., Gorina E., Hopkins P.M., Kardatzke D., Lancaster L. (2014). A phase 3 trial of pirfenidone in patients with idiopathic pulmonary fibrosis. N. Engl. J. Med..

[6] Richeldi L., Azuma A., Cottin V., Kreuter M., Maher T.M., Martinez F.J., Oldham J.M., Valenzuela C., Clerisme-Beaty E., Gordat M. (2025). Nerandomilast in Patients with Idiopathic Pulmonary Fibrosis. N. Engl. J. Med..

[7] Richeldi L., du Bois R.M., Raghu G., Azuma A., Brown K.K., Costabel U., Cottin V., Flaherty K.R., Hansell D.M., Inoue Y. (2014). Efficacy and safety of nintedanib in idiopathic pulmonary fibrosis. N. Engl. J. Med..

[8] Duan F.F., Barron G., Meliton A., Mutlu G.M., Dulin N.O., Schuger L. (2019). P311 Promotes Lung Fibrosis via Stimulation of Transforming Growth Factor-β1, -β2, and -β3 Translation. Am. J. Respir. Cell Mol. Biol..

[9] Okunishi K., DeGraaf A.J., Zasłona Z., Peters-Golden M. (2014). Inhibition of protein translation as a novel mechanism for prostaglandin E2 regulation of cell functions. FASEB J..

[10] Wettlaufer S.H., Penke L.R., Okunishi K., Peters-Golden M. (2017). Distinct PKA regulatory subunits mediate PGE(2) inhibition of TGFβ-1-stimulated collagen I translation and myofibroblast differentiation. Am. J. Physiol. Lung Cell. Mol. Physiol..

[11] Al-Habeeb F., Aloufi N., Traboulsi H., Liu X., Nair P., Haston C., Azuelos I., Huang S.K., White E.S., Gallouzi I.E. (2021). Human antigen R promotes lung fibroblast differentiation to myofibroblasts and increases extracellular matrix production. J. Cell. Physiol..

[12] Chen W., Pilling D., Gomer R.H. (2023). The mRNA-binding protein DDX3 mediates TGF-β1 upregulation of translation and promotes pulmonary fibrosis. JCI Insight.

[13] Adams T.S., Schupp J.C., Poli S., Ayaub E.A., Neumark N., Ahangari F., Chu S.G., Raby B.A., DeIuliis G., Januszyk M. (2020). Single-cell RNA-seq reveals ectopic and aberrant lung-resident cell populations in idiopathic pulmonary fibrosis. Sci. Adv..

[14] Habermann A.C., Gutierrez A.J., Bui L.T., Yahn S.L., Winters N.I., Calvi C.L., Peter L., Chung M.I., Taylor C.J., Jetter C. (2020). Single-cell RNA sequencing reveals profibrotic roles of distinct epithelial and mesenchymal lineages in pulmonary fibrosis. Sci. Adv..

[15] Reyfman P.A., Walter J.M., Joshi N., Anekalla K.R., McQuattie-Pimentel A.C., Chiu S., Fernandez R., Akbarpour M., Chen C.I., Ren Z. (2019). Single-Cell Transcriptomic Analysis of Human Lung Provides Insights into the Pathobiology of Pulmonary Fibrosis. Am. J. Respir. Crit. Care Med..

[16] Duan Q., Reid S.P., Clark N.R., Wang Z., Fernandez N.F., Rouillard A.D., Readhead B., Tritsch S.R., Hodos R., Hafner M. (2016). L1000CDS(2): LINCS L1000 characteristic direction signatures search engine. npj Syst. Biol. Appl..

[17] Lamb J., Crawford E.D., Peck D., Modell J.W., Blat I.C., Wrobel M.J., Lerner J., Brunet J.P., Subramanian A., Ross K.N. (2006). The Connectivity Map: Using gene-expression signatures to connect small molecules, genes, and disease. Science.

[18] Pilarczyk M., Fazel-Najafabadi M., Kouril M., Shamsaei B., Vasiliauskas J., Niu W., Mahi N., Zhang L., Clark N.A., Ren Y. (2022). Connecting omics signatures and revealing biological mechanisms with iLINCS. Nat. Commun..

[19] Subramanian A., Narayan R., Corsello S.M., Peck D.D., Natoli T.E., Lu X., Gould J., Davis J.F., Tubelli A.A., Asiedu J.K. (2017). A Next Generation Connectivity Map: L1000 Platform and the First 1,000,000 Profiles. Cell.

[20] Li A., Chen J.Y., Hsu C.L., Oyang Y.J., Huang H.C., Juan H.F. (2022). A Single-Cell Network-Based Drug Repositioning Strategy for Post-COVID-19 Pulmonary Fibrosis. Pharmaceutics.

[21] Parolo S., Mariotti F., Bora P., Carboni L., Domenici E. (2023). Single-cell-led drug repurposing for Alzheimer’s disease. Sci. Rep..

[22] Zhang X., Liu H., Hock T., Thannickal V.J., Sanders Y.Y. (2013). Histone deacetylase inhibition downregulates collagen 3A1 in fibrotic lung fibroblasts. Int. J. Mol. Sci..

[23] Sanders Y.Y., Hagood J.S., Liu H., Zhang W., Ambalavanan N., Thannickal V.J. (2014). Histone deacetylase inhibition promotes fibroblast apoptosis and ameliorates pulmonary fibrosis in mice. Eur. Respir. J..

[24] Chen L., Alam A., Pac-Soo A., Chen Q., Shang Y., Zhao H., Yao S., Ma D. (2021). Pretreatment with valproic acid alleviates pulmonary fibrosis through epithelial-mesenchymal transition inhibition in vitro and in vivo. Lab. Investig..

[25] Korfei M., Skwarna S., Henneke I., MacKenzie B., Klymenko O., Saito S., Ruppert C., von der Beck D., Mahavadi P., Klepetko W. (2015). Aberrant expression and activity of histone deacetylases in sporadic idiopathic pulmonary fibrosis. Thorax.

[26] Vannan A., Lyu R., Williams A.L., Negretti N.M., Mee E.D., Hirsh J., Hirsh S., Hadad N., Nichols D.S., Calvi C.L. (2025). Spatial transcriptomics identifies molecular niche dysregulation associated with distal lung remodeling in pulmonary fibrosis. Nat. Genet..

[27] Franzen L., Olsson Lindvall M., Huhn M., Ptasinski V., Setyo L., Keith B.P., Collin A., Oag S., Volckaert T., Borde A. (2024). Mapping spatially resolved transcriptomes in human and mouse pulmonary fibrosis. Nat. Genet..

[28] McFarland J.M., Paolella B.R., Warren A., Geiger-Schuller K., Shibue T., Rothberg M., Kuksenko O., Colgan W.N., Jones A., Chambers E. (2020). Multiplexed single-cell transcriptional response profiling to define cancer vulnerabilities and therapeutic mechanism of action. Nat. Commun..

[29] Aibar S., Gonzalez-Blas C.B., Moerman T., Huynh-Thu V.A., Imrichova H., Hulselmans G., Rambow F., Marine J.C., Geurts P., Aerts J. (2017). SCENIC: Single-cell regulatory network inference and clustering. Nat. Methods.

[30] Hu M., Che P., Han X., Cai G.Q., Liu G., Antony V., Luckhardt T., Siegal G.P., Zhou Y., Liu R.M. (2014). Therapeutic targeting of SRC kinase in myofibroblast differentiation and pulmonary fibrosis. J. Pharmacol. Exp. Ther..

[31] Abdalla M., Thompson L., Gurley E., Burke S., Ujjin J., Newsome R., Somanath P.R. (2015). Dasatinib inhibits TGFβ-induced myofibroblast differentiation through Src-SRF Pathway. Eur. J. Pharmacol..

[32] Yilmaz O., Oztay F., Kayalar O. (2015). Dasatinib attenuated bleomycin-induced pulmonary fibrosis in mice. Growth Factors.

[33] Mercer P.F., Woodcock H.V., Eley J.D., Platé M., Sulikowski M.G., Durrenberger P.F., Franklin L., Nanthakumar C.B., Man Y., Genovese F. (2016). Exploration of a potent PI3 kinase/mTOR inhibitor as a novel anti-fibrotic agent in IPF. Thorax.

[34] Peyvandipour A., Saberian N., Shafi A., Donato M., Draghici S. (2018). A novel computational approach for drug repurposing using systems biology. Bioinformatics.

[35] Meijles D.N., Cull J.J., Cooper S.T., Markou T., Hardyman M.A., Fuller S.J., Alharbi H.O., Haines Z.H., Alcantara-Alonso V., Glennon P.E. (2021). The anti-cancer drug dabrafenib is not cardiotoxic and inhibits cardiac remodelling and fibrosis in a murine model of hypertension. Clin. Sci..

[36] Shi Y., Huang C., Yi H., Cao Q., Zhao Y., Chen J., Chen X., Pollock C. (2020). RIPK3 blockade attenuates kidney fibrosis in a folic acid model of renal injury. FASEB J..

[37] Falchook G.S., Long G.V., Kurzrock R., Kim K.B., Arkenau H.-T., Brown M.P., Hamid O., Infante J.R., Millward M., Pavlick A. (2014). Dose selection, pharmacokinetics, and pharmacodynamics of BRAF inhibitor dabrafenib (GSK2118436). Clin. Cancer Res..

[38] Ouellet D., Gibiansky E., Leonowens C., O’Hagan A., Haney P., Switzky J., Goodman V.L. (2014). Population pharmacokinetics of dabrafenib, a BRAF inhibitor: Effect of dose, time, covariates, and relationship with its metabolites. J. Clin. Pharmacol..

[39] Blair A., Zmuda F., Malviya G., Tavares A.A.S., Tamagnan G.D., Chalmers A.J., Dewar D., Pimlott S.L., Sutherland A. (2015). A novel 18F-labelled high affinity agent for PET imaging of the translocator protein. Chem. Sci..

[40] Seneviratne M.S.D., Faccenda D., De Biase V., Campanella M. (2012). PK11195 inhibits mitophagy targeting the F1Fo-ATPsynthase in Bcl-2 knock-down cells. Curr. Mol. Med..

[41] Tu L.N., Zhao A.H., Stocco D.M., Selvaraj V. (2015). PK11195 effect on steroidogenesis is not mediated through the translocator protein (TSPO). Endocrinology.

