# Supplementary material for: Single-Cell Transcriptomic Analysis Identifies a Translation/RBP-Related Fibroblast Signature for Drug Repurposing in Idiopathic Pulmonary Fibrosis
Source: Int J Mol Sci. 2026 Oct 30;27(21):9736. doi: 10.3390/ijms27219736 (PMC13762596; doi:10.3390/ijms27219736)
Supplement: Supplementary file 1 [file ijms-27-09736-s001.zip › Supplementary figures and figure legends.pdf]

Single-Cell Transcriptomic Analysis Identifies a Translation/RBP-Related Fibroblast Signature for Drug Repurposing in Idiopathic Pulmonary Fibrosis

Yongkang Qiao [1,†], Chaoyue Cui [1,†], Yanhong Han [1], Xinxin Tang [1,2], Zhen Yang [1,2], Chuang Ge [1,*]

Supplementary Figures and figure legends

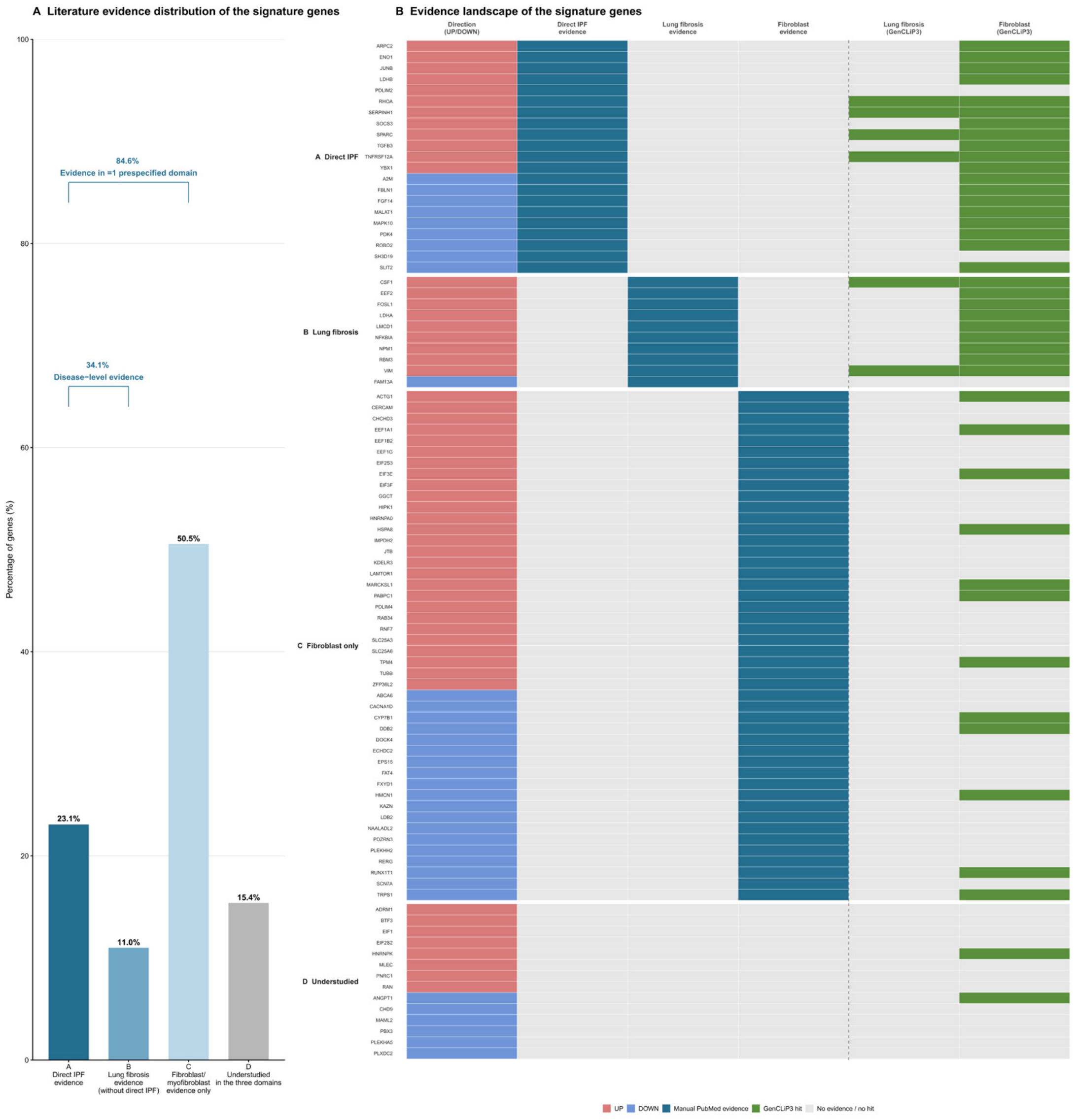

Supplementary Figure S1. Literature evidence for signature genes in IPF, lung fibrosis, and fibroblast-related research.

(A) Literature-evidence classification of the signature genes based on gene-level PubMed review. Genes were assigned to four categories: A, direct IPF evidence; B, lung fibrosis evidence without confirmed direct IPF evidence; C, fibroblast/myofibroblast-related evidence only; and D, relatively understudied across the three prespecified domains. Overall, 84.6% of the genes had evidence in at least one prespecified domain, whereas 34.1% had disease-level evidence in IPF or lung fibrosis.

(B) Gene-level evidence landscape for the signature genes. Each row represents one gene and shows its UP/DOWN direction together with PubMed evidence identified by gene-level review in direct IPF, lung fibrosis, and fibroblast-related research, as well as GenCLiP3 text-mining hits for lung fibrosis and fibroblast-related terms. Blue indicates PubMed evidence identified by gene-level review, green indicates a GenCLiP3 text-mining hit, and gray indicates no corresponding evidence or no hit. The A–D evidence tiers were defined on the basis of gene-level PubMed review, whereas GenCLiP3 results are shown as independent complementary text-mining evidence.

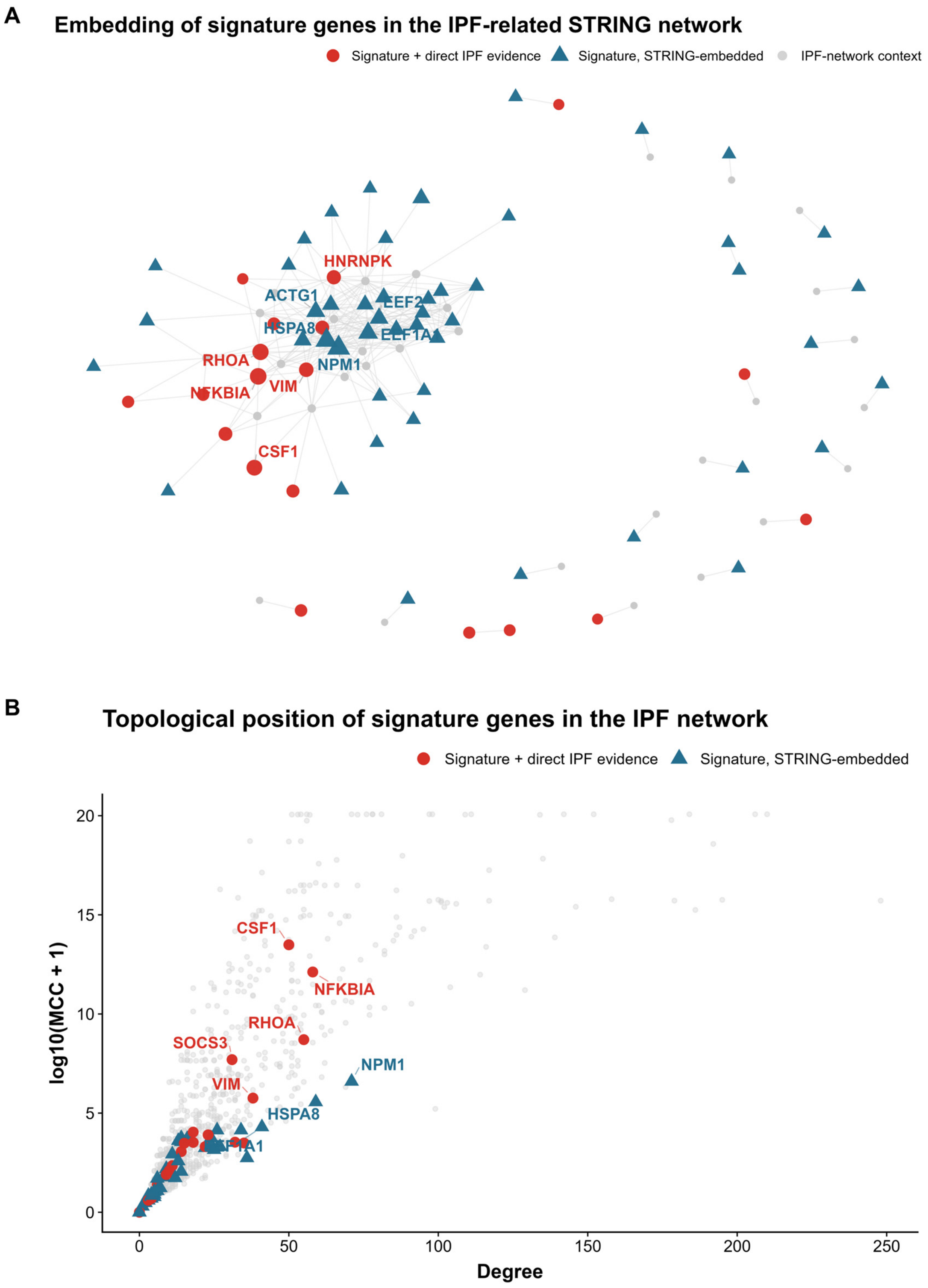

Supplementary Figure S2. Embedding and topological characteristics of signature genes in the IPF-related STRING network.

(A) Embedding of signature genes within the IPF-related STRING functional interaction network. Red circles indicate signature genes with direct DisGeNET IPF annotation, blue triangles indicate signature genes without direct DisGeNET IPF annotation but embedded in the network through STRING interactions, and gray nodes indicate IPF-network context nodes used to visualize the surrounding

interaction structure. For clarity, a signature-centered network context is shown, whereas all topological metrics were calculated using the complete 1,496-node STRING network.

(B) Distribution of Degree and MCC for signature genes within the complete IPF-related network. Gray points represent background nodes in the full network, red circles represent signature genes with direct IPF evidence, and blue triangles represent STRING-embedded signature genes. The x-axis indicates Degree and the y-axis indicates log10(MCC + 1). Labeled genes represent selected signature genes with relatively high Degree or MCC.

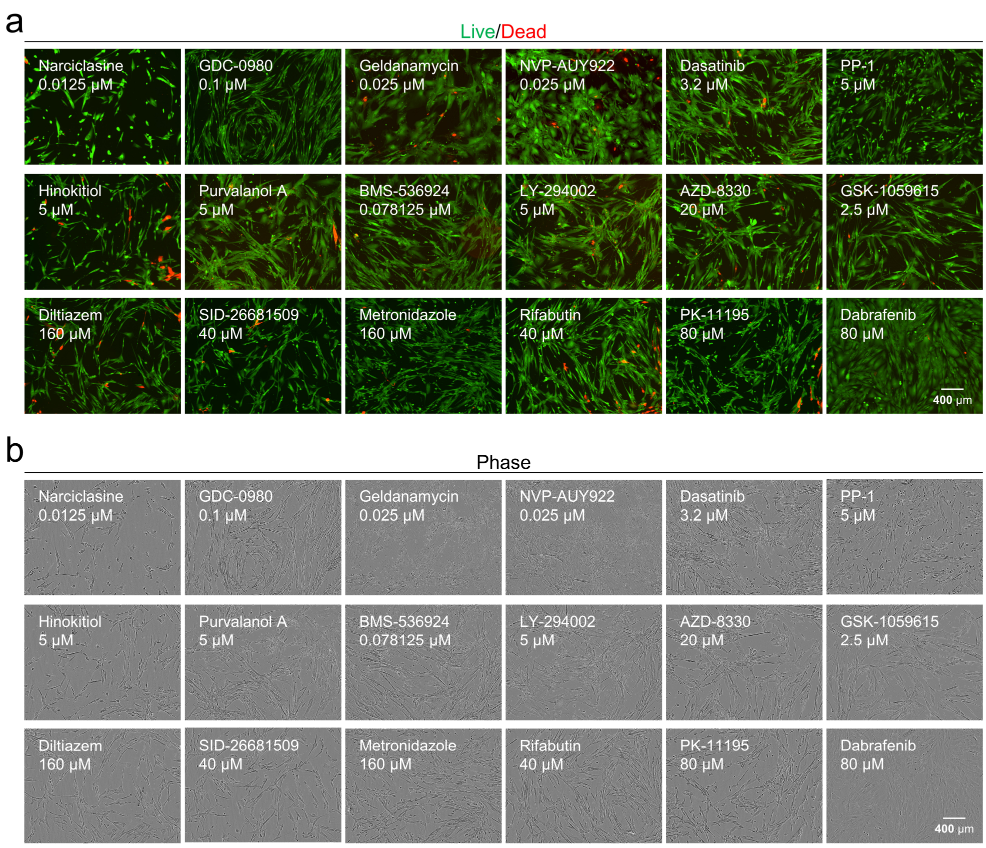

Supplementary Figure S3. Cellular tolerance of candidate compounds in HFL-1 cells assessed by Live/Dead staining and phase-contrast imaging.

(a) Representative Live/Dead fluorescence images of HFL-1 cells treated with the indicated compounds and concentrations. Live cells were labeled with Calcein-AM (green), and dead cells were labeled with propidium iodide (PI; red). (b) Corresponding phase-contrast images showing cell density and morphology under the same treatment conditions. These imaging assessments were considered together with the CCK-8 viability results when selecting concentrations for subsequent phenotypic evaluation. Scale bars, 400 μm.
